# Supplementary figures and images for: LAMP3 deficiency affects surfactant homeostasis in mice
Source: PLoS Genet. 2021 Jun 23;17(6):e1009619. doi: 10.1371/journal.pgen.1009619 (PMC8259984; doi:10.1371/journal.pgen.1009619)

**A**

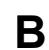

Supplement: S1 Fig — (A) Two-dimensional principal component analysis (PCA) loadings plot of the BAL lipidome of wild type (black) and Lamp3-/- (green) mice. Each point represents one individual animal. Ellipses show the 95% confidence interval of the group. PC1 and PC2 explain 54.1% of the variability in the data set. Confidence intervals show a complete overlapping of samples belonging to both groups. Both groups are not separated by PCA. (B) Hierarchical clustering of 158 lipid species (of 393 after application of 90% occupation threshold) identified in eight wildtype and eight Lamp3-/- mice lung tissue samples. Each row represents a lipid species and each column a sample. (C) The hierarchical clustered tree of sample groups is not able to correlate both samples into two distinct branches. (PDF) [file pgen.1009619.s002.pdf]

S2 Fig

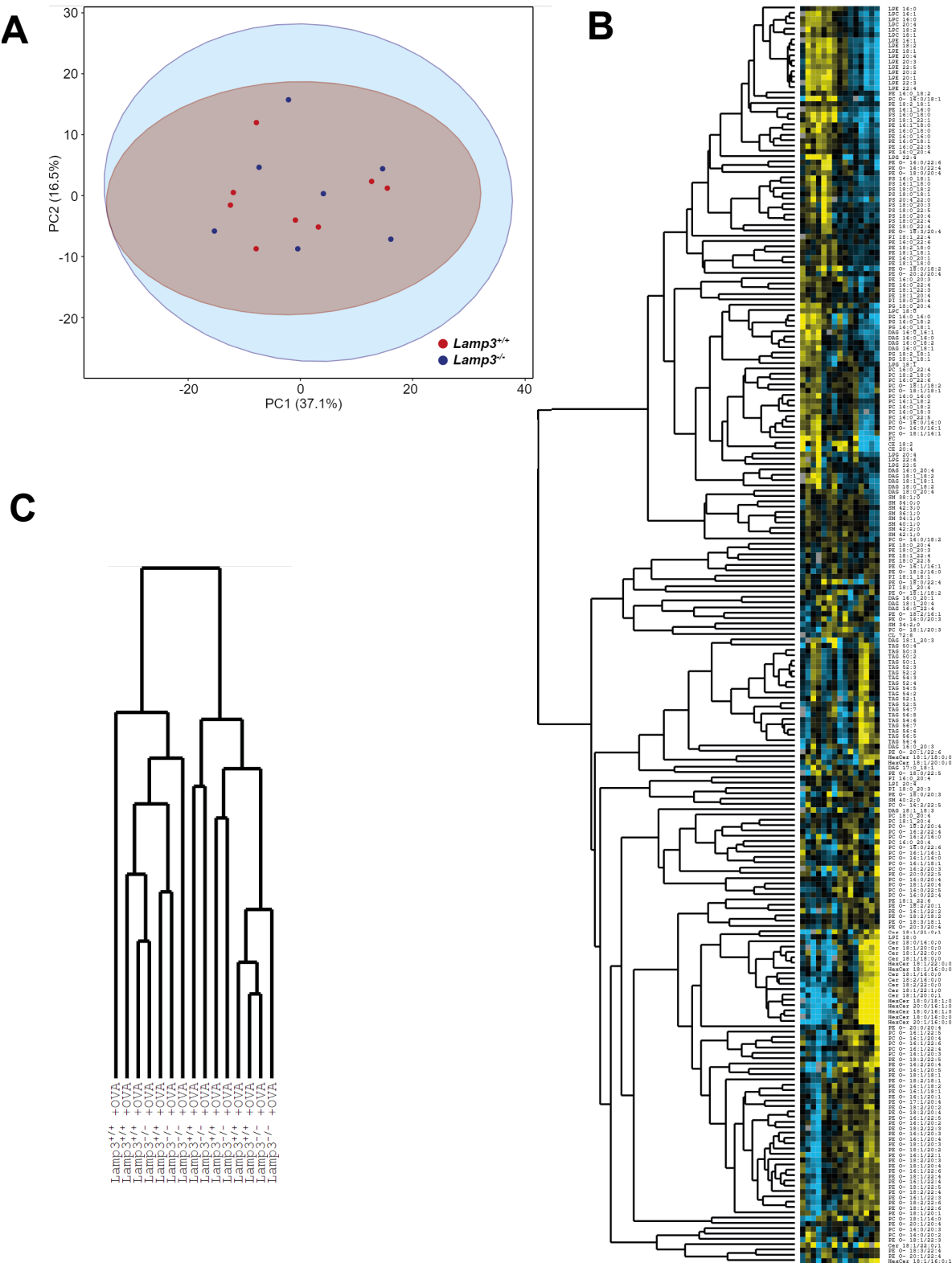

Supplement: S2 Fig — (A) Two-dimensional principal component analysis (PCA) loadings plot of the BAL lipidome of wild type (blue) and Lamp3-/- (red) mice. Each point represents one individual animal. Ellipses show the 95% confidence interval of the group. PC1 and PC2 explain 53.6% of the variability in the data set. Confidence intervals show a complete overlapping of samples belonging to both groups. No separation of samples into two distinct groups is performed by PCA. (B) Hierarchical clustering of 237 lipid species (of 394 after a threshold of 90% occupation was applied) identified in eight wildtype and seven Lamp3-/- mice lung tissue samples. Each row represents a lipid species and each column a sample. (C) The hierarchical clustering analysis clusters samples into multiple branches not correlating to the distinct groups. (PDF) [file pgen.1009619.s003.pdf]

S3 Fig

**A**

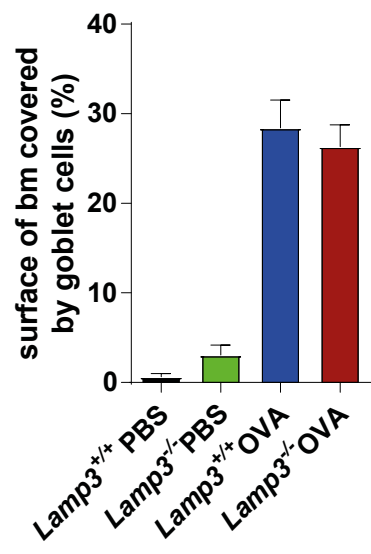

**B**

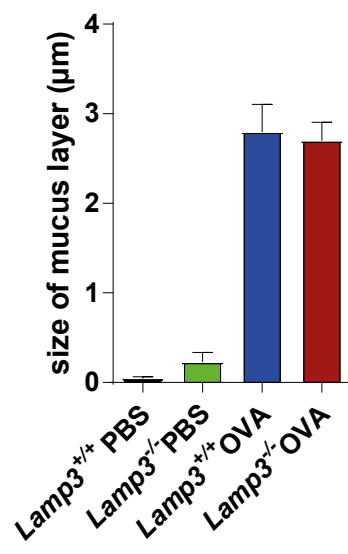

**C**

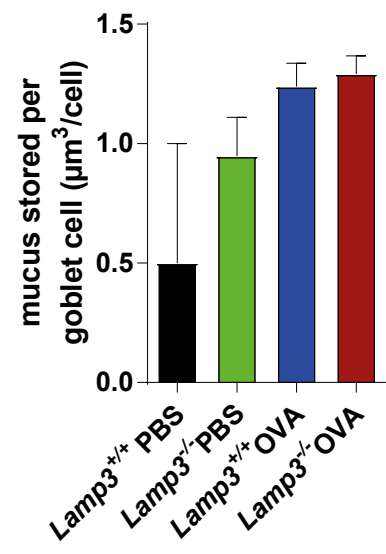

Supplement: S3 Fig — Quantification of mucus production in airway epithelial cells. OVA-induced experimental asthma increases mucus production. Lamp3-/- phenotype does not alter the amount and distribution of cells stained positive for mucus. Area of epithelial basal membrane covered by goblet cells (A), stored mucus volume per basal membrane area (B), and mucus stored per goblet cell (C). A-C: n = 8 mice per group. (PDF) [file pgen.1009619.s004.pdf]

**S4 Fig**

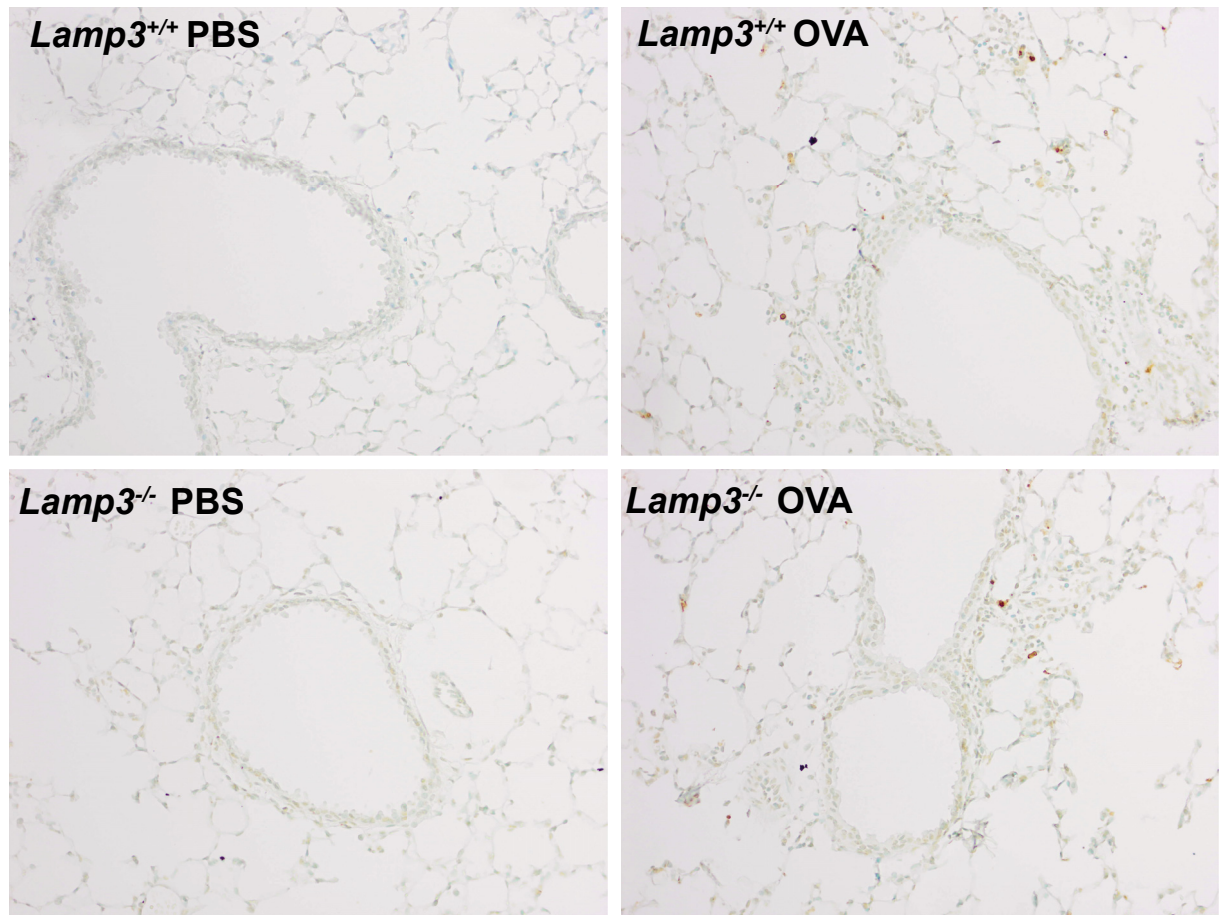

Supplement: S4 Fig — Apoptosis staining (TUNEL assay) of inflated formalin-fixed paraffin embedded crosssections of the lung. Lamp3-/- phenotype does not alter the amount and distribution of cells stained positive for apoptosis (brown staining). Representative staining of healthy wildtype (upper left), healthy knockout Lamp3-/- (lower left), asthmatic wildtype (upper right), and asthmatic knockout Lamp3-/- (lower right). (PDF) [file pgen.1009619.s005.pdf]

S5 Fig

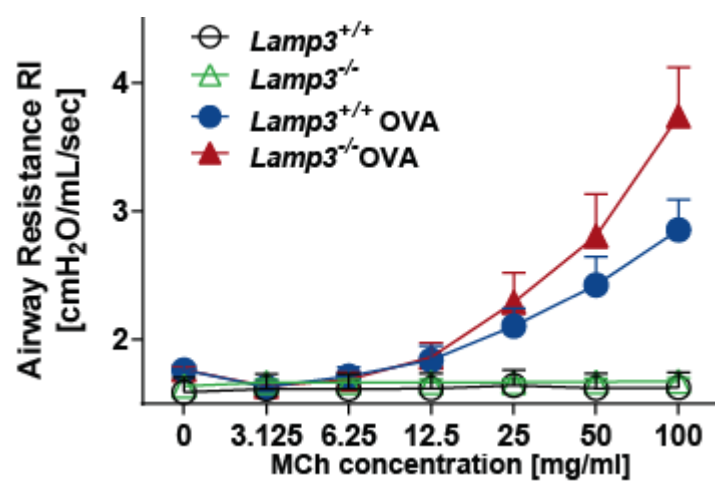

Supplement: S5 Fig — Increase in airway resistance of the four experimental groups (wildtype, Lamp3-/-, wildtype + OVA, Lamp3-/- + OVA). N = 8 (per genotype) in absolute values, significances calculated for methacholine provocation test after 100 mg/ml methacholine exposure, ns = not significant; 0.05; **** = p ≤ 0.0001. (PDF) [file pgen.1009619.s006.pdf]
